# Creating an Agenda for Black Birth Equity: Black Voices Matter

**DOI:** 10.1089/heq.2021.0156

**Published:** 2023-03-14

**Authors:** Sharla Smith, Michelle Redmond, Sierra Stites, Jaleen Sims, Megha Ramaswamy, Patricia J. Kelly

**Affiliations:** ^1^ Population Health, University of Kansas School of Medicine, Kansas City, Kansas, USA.; ^2^ Population Health, University of Kansas School of Medicine, Wichita, Kansas, USA.; ^3^ Department of Obstetrics and Gynecology, Jackson-Hinds Comprehensive Health Center Jackson, Mississippi, USA.; ^4^ Retired-School of Nursing, University of Missouri-Kansas City, Kansas City, Missouri, USA.

**Keywords:** Birth Equity, Equity, Health Disparities, Maternal and Child Health, Voices

## Abstract

**Background:**

The grim inequity that Black women and infants are more than twice as likely to die during birth than their white counterparts is a public health crisis.

**Methods:**

Guided by principles of critical race theory, we used content analysis to analyze the themes of the presentation made by five Black community members on a 2020 Juneteenth panel, a holiday celebrating the emancipation of those who had been enslaved in the United States.

**Results:**

Panelists sparked the conversation by unpacking the traumatic experiences of health inequities and structural racism on Black families and diverse caregivers. As a part of qualitative content analysis, four major themes emerged: (1) the matrix of domination, (2) specific oppressions of the health care system, (3) empowerment reconceptualized, and (4) dimensions of racism. Participants also discussed how racial disparities may have exacerbated the complexities and challenges of elevating Black voices and creating birth equity.

**Discussion:**

Based on Black families' experiences, four areas must be addressed: health care system's policies of oppression that create barriers to listening to Black women, reconceptualizing retention for providers of color and support for Black women and their families, and racism.

## Background

Every day in the United States, 700 women die of pregnancy-related complications; 22,335 Black infants die before the age of one.^1^ For an industrialized, 21st century nation, these are unacceptable and tragic inequities that are the result of interpersonal and structural racism that is omnipresent throughout the health care system and our communities.^2^

Interpersonal racism is defined as misinformation and stereotypes toward another group and perform an act of harassment, exclusion, marginalization, discrimination, hate, or violence.^3^ Structural racism is a normalization of systems, policies, and practices that are historical and cultural, and work together that create inequality for people of color such as racism.^2,3^ These practices can be directly tied to health inequalities such as infant and maternal mortality and are well described as the part of an iceberg that is beneath the water—unseen, but dangerous and hard to eliminate.^4^

One key part of this racism is that we, as a society and as a health care system do not listen to Black women.^5^ Complaints about pain and pregnancy complications by Black patients are disregarded more than for White patients.^6^ Fame and education are no guarantee of being heard from tennis star Serena Williams, to Grammy winner Beyonce, to Pediatric Chief Resident Chaniece Wallace, the inattention persists. While Beyonce and Serena both survived nearly fatal pregnancy complications shortly after emergency C-sections, Chaniece died after giving birth despite insisting on care. For Black women, childbirth is inherently risky—the reality is that you cannot educate or access your way out of danger.

### Critical race theory

Critical race theory, created in the field of law, centers on examining and fixing inequities around race, racism, and power.^7^ Using critical race theory (CRT) to unpack the issues faced by Black women during childbirth can create possible solutions for better health outcomes. Ford and Airhihenbuwa offer an adaption of constructs from the original CRT suitable for public health: *race consciousness*, *contemporary mechanisms*, *centering in the margins*, and *praxis.*^8^ To explain these constructs, we place them in the framework of Black women and maternal health. *Race consciousness* means capturing from Black women their clear understanding of the social context of race and racism*. Contemporary mechanisms* refer to the everyday experience of racism in society directly linked to Black women. *Centering in the margin*s makes the perspective of Black women the central focus of discussion on birth inequity issues. *Praxis* is understanding the connection of theory, research, and community practice/engagement of Black women in their work to create solutions to birth inequity.

For Black women seeking care through the U.S. health system, using the lens of CRT can help frame their experiences away from their race as a risk factor and toward their experiences within the health care system because of racism. Ford and Airhihenbuwa state it best, “…race is socially constructed it is less a risk factor itself than a marker of risk for racism-related exposures. Race is useful in that it enables the identification of persons at risk for exposures that vary by racial category (e.g., discrimination).”^8^ This understanding captures the experiences of women in our panel.

The goal of this article, the results of a public presentation to acknowledge Juneteenth, is to bring the voices of community members of color into the academic literature and hear specifics of traumatic experiences of health and structural inequities in our community.

## Methods

We analyzed the transcript of a panel discussion of Black providers and consumers about their perspectives on maternal/neonatal mortality. The panel was available on a zoom platform and viewed by 1600 individuals, who were able to ask questions and make comments.

### Participants

The panel was organized by the Kansas Birth Equity Network (KBEN), an organization launched by the Kansas Sisters and Brothers for Healthy Infants Initiative (SBHI), a partnership between two academic researchers, and members of Black Greek Letter Organizations (BGLO) in Wichita, Kansas. The KBEN is a unified patient engagement program of patients and diverse stakeholders (*n*=44) working together with academics to create solutions that improve Black maternal, paternal, and neonatal health in Kansas through training, research, health care, and advocacy. The network's objectives are to train stakeholders of color on birth equity, elevate the voices of Black mothers and fathers, increase patient activation in research, health care, and advocacy, and create a 5-year research agenda to reduce maternal/neonatal mortality.

The panel consisted of two Black Kansas community activists/mothers, one Black father, a Black Obstetric and Gynecology, and an Afro-Latina doula.

### Analysis

Guided by a CRT and Black feminist theory, we used content analysis to analyze themes of the presentations.^7,8^ The transcript from the 90-min event was transcribed verbatim and coded by two independent investigators experienced in qualitative research. Codes were reviewed with the principal investigator/author who used the codes to identify themes and relevant quotes. Themes and quotes were reviewed with all authors to arrive at a consensus about whether the material reflected the event and the important messages to be communicated to a broader audience.

## Results

We identified four major themes: *the matrix of domination*, *specific oppressions of the health care system*, *dimensions of racism,* and *empowerment reconceptualized* (Table 1).

**Table 1.** Black Birth Equity Themes

Themes	Quotes
The matrix of domination oppression had two subthemes: Origins and Today	Words from the subtheme of *Origins* made clear panelists' understanding of the way and how of oppression: – Guess where it starts? Not when you get pregnant. Middle school, elementary school. Our babies got stress in their belly. [They are] on fight or flight all the time. You think that turns out just because she figured out she got pregnant? Just because he figured out he's going to be a father? Absolutely not. – All these factors in society. The preschool to prison pipeline, the racism in the medical system, just everything that Black women face in society, and it destroys the family. That is the long-term cost of this genocide. I won't call it anything else. It is genocide, and I don't want to talk about it in economic dollars. It is a human cost, and these are not numbers. These are mothers, these are babies. —- Just that wear and tear on our bodies; the systemic racism that we have to encounter, the lack of access to healthcare, the lack of quality education for our kids; the disparity in pay parity when we go to work, and we're working just as hard to make just as less. it's not happening in a vacuum. It's these things that are compounding that's rising our blood pressure. And here we are trying to bear children, or come home and take care of children. We're not sleeping. We're stressed, we have heightened anxiety. I worry about him *(points at husband)* all the time. I worry about my kids all the time. I cannot just sit and relax, like some people can. I tell people I am always on guard. It is fight or flight, and that is completely unhealthy for our bodies. – Every time I hear the ambulance or police, I am just terrified, just in trauma, if my son is not next to me because of all the events that are happening. We have to think about and we have to have these conversations about how this is impacting the Black body. The subtheme *Today* made clear the impact: –I am a Black woman under forty with one child, and I dare not have another one less I test the odds. When I started speaking with other women, other Black women, I found that again, our stories were very similar in our care, the quality of that care, and the way in which we play a little game of roulette every time we decide to have a child. – People want us to be polite about our pain … to be polite to our oppressors … to people on our neck literally and figuratively. We're dying and we're apologizing for it. That's stressful. It's stressful to have to choose your language when you're trying to just save your life. – We tell people to be polite when they're contextualizing what racism *feels* like for a person living it. What that does physiologically to the body. – We can't use [the word] bias when it's actually racism. It's the elephant in the room that makes people uncomfortable, but until we can discuss it, we can't face it. we can't change it.
Specific oppression: the health care system had two subthemes: no listening and no consideration (could use a stronger term for the latter) (This needs some work!)	The subtheme of *No listening* was heard again and again. We're not centering Black mothers. We're not listening to them. We're not amplifying their voices. We're not replacing the safety net that could potentially save lives, –Watching my wife go through it, giving birth …the doctor really wasn't paying attention to her. She was telling him, “I have a headache. I don't feel good.” And [the doctor] being dismissive … that's a problem Examples of *No consideration* included: –When I was on the table being cut open by my doctor, a nurse rushed in to stop the procedure to hand me a telephone, to bring a nasty telephone as I laid on the table being cut open to get my child, whose heart rate was dropping, it was an emergency. She brought me a phone to let me know that the procedure could not go on until I had a doctor on file who would take the medical card. —[the nurse] looked at me and my Black partner and asked me where is the dad, and is he a white man. My child is a little more fair-skinned than I, and she just did not know. I think she's miss genetics that training day where we learn about recessive traits and what children can look like. –In births that I've gone to, the nurse will walk in a room and just walk right past the father as if she didn't see him. And it's dehumanizing. And I can't imagine what that would be like, going to the birth of my child and feeling othered, feeling excluded, feeling made to feel like I don't belong there. But could also have a theme of understanding the overall racism. – No matter how early you went to see your doctor, even though that's something you need to do, it's preventative, no matter how on top of things you were, you could still come out dead, regardless of age, social economic status; it is because you are Black in America and our health system, like our other systems, has not decided to remedy historical precedent. –People who started our medical institutions, people who teach our physicians, people who train them, don't train them to talk to you. Don't train them how to treat Black bodies, women in pregnancy. [after giving birth] When a patient presents to a hospital with complaint of headache or abdominal pain, they treat those particular symptoms; however, they overlook those severe range blood pressures meaning blood pressures that are greater than 160/110. They consider them insignificant because she's no longer pregnant. This is an oversight and can lead to significant morbidity and potentially mortality for this patient. In doing reviews of maternal deaths, we've definitely seen this. –I think it really boils down to just disregard and devaluing Black mothers and Black babies. We aren't focusing the funding where we need to. We aren't asking the community what the community needs. We're basically telling folks what they need, and it's tone-deaf, and it needs to stop
Empowerment reconceptualized also had two subthemes, one specific to the health care system and one for the advocates and members of the African American community	Panelists were clear that changes in the *Health care system* had to occur on an individual and a policy level. – We need to intentionally train our health providers and look to measure cultural competency in health systems, –When nurses come in the room, one thing they can do is ask the dad's name. He's sitting right there! Just basic humanity could go a long way. – Every state and hospital should have a maternal morbidity and mortality review committee…When you have these committees, and you can review the morbidities and the deaths among your mothers, it's not a point of pointing the finger at who did what wrong, but it's a point where you can say, “That's where we went wrong in her care.” – Put together what we call “care bundles” that help standardize care so that you hope that the provider bias will be removed from it because you're following a care plan. [put] a system in place where they fall through one crack … you can catch them before they hit the ground. - On the positive side, [there is] more awareness, which is what we're doing, of this matter, and legislation. There are lots of organizations that are going on Capitol Hill and talking to their senators and their representatives about different policies that can be put in place to create systematic changes in hospitals, in care bundles, to help standardize patient care. So, our goal with that legislation is to help us standardize this care, *Advocates and community members* need support in continuing their work. – I will not sit here and pretend that any intervention strategy that does not include the dismantling of white supremacist systems will work. It won't work.—we need to encourage and empower our fathers to be advocates. You don't want to be deemed as the mad Black man in the room over there, but encourage them to be advocates in a way that is respectful, that makes his loved one's voice heard. I don't care if you gotta go call the nurse, the nurse's supervisor, or call the physician to the room. We have to do something to where we empower them to keep speaking up. If they call out and no one comes, they call out again. And if no one comes, they call out again. And there's blood all over the because she's having a postpartum hemorrhage, and no one comes, they call out again. You know what? Walk out the room and go down the hallway, “I need someone to come to this room and assess my girlfriend, my fiancée, my wife, because she is bleeding, and she looks different than what I'm used to.” – We have got to start using our own tools to dismantle the system because there is no other way for us to stop this train that is moving in this direction. We just got to take it up off these tracks. I think this means being radical about a number of things: our self-care; our self-preservation; the way we work in communities; the way we see each other; our ubuntu; the way we treat each other with humanity; the way we treat our communities; –Keep calling out the elephant in the room and call a spade a spade. Acknowledge what racism is doing. It's killing people. –The path forward is keep talking. Keep calling out when these things happen. Keep calling out when doctors and health care providers aren't listening and aren't taking Black women serious, not taking their health serious. Whether it is physical in this instance or it is mental. We have to keep calling them out. Black fathers have to be, we have to be involved. We have to be there. We have to stand there for our women, stand there for our children, our future children, everybody. We just have to keep being involved, keep being in the community, keep being in these doctors' offices, in the labor and delivery rooms, just everywhere. We just have to keep being present until these changes come about. We have to talk about our stories. We have to all be at the table, and especially Black women. We cannot discount Black women's voices. It's not fair. Black women deserve the biggest seat at the table on this issue. –Keep calling out the elephant in the room and call a spade a spade. Acknowledge what racism is doing. It's killing people.

The first theme, *matrix of domination,* had two subthemes: *origins* and *today's manifestations*. Words from the subtheme *origins* made clear panelists' understanding oppression:

Guess where it starts? Not when you get pregnant. Middle school, elementary school. Our babies got stress in their belly. [They are] on fight or flight all the time. You think that turns out just because she figured out she got pregnant? Just because he figured out he's going to be a father? Not.

All these factors in society. The preschool to prison pipeline, the racism in the medical system, just everything that Black women face in society, and it destroys the family. That is the long-term cost of this genocide. I won't call it anything else. It is genocide, and I don't want to talk about it in economic dollars. It is a human cost, and these are not numbers. These are mothers, these are babies.

Just that wear and tear on our bodies; the systemic racism that we encounter, the lack of access to healthcare, the lack of quality education for our kids; the disparity in pay parity when we go to work, and we're working just as hard to make just as less. it's not happening in a vacuum. It's these things that are compounding that's raising our blood pressure. And here we are trying to bear children or come home and take care of children. We're not sleeping. We're stressed, we have heightened anxiety. I worry about him [husband) all the time. I worry about my kids all the time. I cannot just sit and relax like some people can. I tell people I am always on guard. It is fight or flight, and that is completely unhealthy for our bodies.

Every time I hear the ambulance or police, I am just terrified, just in trauma, if my son is not next to me because of all the events that are happening. We have to think about, and we have to have these conversations about how this is impacting the Black body.

The subtheme, *today's manifestations,* made clear the current impact:

I am a Black woman under forty with one child, and I dare not have another one less I test the odds. When I started speaking with other Black women, I found that again, our stories were very similar in our care, the quality of that care, and the way in which we play a little game of roulette every time we decide to have a child.

People want us to be polite about our pain … to be polite to our oppressors … to people on our neck literally and figuratively. We're dying and we're apologizing for it. That's stressful. It's stressful to have to choose your language when you're trying to just save your life.

We tell people to be polite when they're contextualizing what racism *feels* like for a person living it. What does that do physiologically to the body?

We use [the word] bias when it's actually racism. It's the elephant in the room that makes people uncomfortable, but until we can discuss it, we can't face it. we can't change it.

The second theme, *specific oppression of the health care system,* had two subthemes: *not listening* and *no consideration*. The subtheme of *not listening* was heard repeatedly.

We're not centering Black mothers. We're not listening to them. We're not amplifying their voices. We're not replacing the safety net that could potentially save lives,

Watching my wife go through giving birth …the doctor really wasn't paying attention to her. She was telling him, “I have a headache. I don't feel good.” And [the doctor] being dismissive … that's a problem.

Examples of *no consideration* included:

When I was on the table being cut open by my doctor, a nurse rushed in to stop the procedure to hand me a telephone, to bring a nasty telephone as I laid on the table being cut open to get my child, whose heart rate was dropping, it was an emergency. She brought me a phone to let me know that the procedure could not go on until I had a doctor on file who would take the medical card.

[The nurse] looked at me and my Black partner and asked me where is the dad, and is he a white man. My child is a little more fair-skinned than I, and she just did not know. I think she had missed genetics that training day where we learn about recessive traits and what children can look like.

In births that I've gone to, the nurse will walk in a room and just walk right past the father as if she didn't see him. And it's dehumanizing. And I can't imagine what that would be like, going to the birth of my child and feeling othered, feeling excluded, feeling made to feel like I don't belong there.

Examples of the third theme, *dimensions of racism,* include:

No matter how early you went to see your doctor, even though that's something you need to do, no matter how on top of things you were, you could still come out dead, regardless of age, social economic status. It is because you are Black in America.

People who started our medical institutions, people who teach our physicians, people who train them, don't train them to talk to you, don't train them how to treat Black bodies, women in pregnancy.

[after giving birth] When a patient presents to a hospital with complaint of headache or abdominal pain, they treat those particular symptoms; however, they overlook say, severe blood pressures greater than 160/110—them insignificant because she's no longer pregnant. This is an oversight and can lead to significant morbidity and potentially mortality for this patient. In doing reviews of maternal deaths, we've definitely seen this.

I think it really boils down to just disregard and devaluing Black mothers and Black babies. We aren't focusing the funding where we need to. We aren't asking the community what the community needs. We're basically telling folks what they need, and it's tone-deaf, and it needs to stop.

The fourth theme, *empowerment reconceptualized* also had two subthemes, one specific to the health care system and one for the advocates and members of the Black community. Panelists were clear that changes in the *health care system* had to occur on an individual and a policy level.

We need to intentionally train our health providers and look to measure cultural competency in health systems.

When nurses come in the room, one thing they can do is ask the dad's name. He's sitting right there! Just basic humanity could go a long way.

Every state and hospital should have a maternal morbidity and mortality review committee…When you have these committees, and you can review the morbidities and the deaths among your mothers, it's not a point of pointing the finger at who did what wrong, but it's a point where you can say, “That's where we went wrong in her care.”

Put together what we call ‘care bundles' that help standardize care so that you hope that the provider bias will be removed from it because you're following a care plan. [put] a system in place where they fall through one crack … you can catch them before they hit the ground.

On the positive side, [there is] more awareness, which is what we're doing, of this matter, and legislation. There are lots of organizations that are on Capitol Hill, talking to their senators and their representatives about different policies that can be put in place to create systematic changes in hospitals, in care bundles, to help standardize patient care. So, our goal with that legislation is to help us standardize this care.

A second subtheme here, *advocates and members of the Black community need support* in continuing their work.

I will not sit here and pretend that any intervention strategy that does not include the dismantling of white supremacist systems will work. It won't work. We need to encourage and empower our fathers to be advocates. You don't want to be deemed as the mad Black man in the room over there but encourage them to be advocates in a way that is respectful, that makes his loved one's voice heard. I don't care if you gotta go call the nurse, the nurse's supervisor, or the physician to the room. We have to do something to where we empower them to keep speaking up. If they call out and no one comes, they call out again. And if no one comes, they call out again. And there's blood all over the because she's having a postpartum hemorrhage, and no one comes, they call out again. You know what? Walk out the room and go down the hallway, “I need someone to come to this room and assess my girlfriend, my fiancée, my wife, because she is bleeding, and she looks different than what I'm used to.”

We have got to start using our own tools to dismantle the system because there is no other way for us to stop this train that is moving in this direction. We just got to take it up off these tracks. I think this means being radical about a number of things: our self-care; our self-preservation; the way we work in communities; the way we see each other; our ubuntu; the way we treat each other with humanity; the way we treat our communities.

Keep calling out the elephant in the room and call a spade a spade. Acknowledge what racism is doing. It's killing people.

The path forward is to keep talking. Keep calling out when these things happen. Keep calling out when doctors and health care providers aren't listening and aren't taking Black women seriously, not taking their health seriously. Whether it is physical in this instance or it is mental. We have to keep calling them out. Black fathers have to be, we have to be involved. We have to be there. We have to stand there for our women, stand there for our children, our future children, everybody. We just have to keep being involved, keep being in the community, keep being in these doctors' offices, in the labor and delivery rooms, just everywhere. We just have to keep being present until these changes come about. We have to talk about our stories. We have to all be at the table and especially Black women. We cannot discount Black women's voices.

## Discussion

Findings from our panel discussion emphasize the magnitude of the effect of structural racism on Black women's experiences during pregnancy and delivery. Our results indicate Black women feel ignored in their interactions with health care providers. They feel burdened by a history of racism that affects their health and that of their unborn child and they want to see changes in obstetric care to reduce health inequity.

Our findings on Black women's health care experiences, particularly on maternal health inequalities, structural racism, oppression, and empowerment, can be understood through CRT.^7^ Our results build upon previous work suggesting that fear of disrespect and abuse and loss of autonomy are drivers for planned unattended home births and reduce uptake of care even among Black women with known risk factors.^9^ Too afraid to go, fears of dignity violations, and fear of not being listened to are reasons for absent or limited prenatal care health services in the United States.

All participants highlighted the need for health care system change, listening to women, and dismantling racism, all recommended in prior studies. Similarly, research shows that Black women are not listened to, prefer providers of colors, and acknowledge that racism is a public health problem.^5^ For example, over 30 Black women living in the western United States interviewed about their experiences found medical care to be an issue of structural racism, including inadequate prenatal care and communication with providers.^10^

This study was limited to a focus on one state and a small number of participants in a virtual panel. It does not represent the Black community or health care organizations. Future research, including similar stakeholders, may reveal different or additional results in experiences and approaches to reduce maternal traumatic experiences, health inequities, and structural racism.

### Health equity implications

The persistent state of maternal and child health disparities in Kansas has profound implications for the overall health of Black mothers, fathers, and children. Creating an agenda for birth equity that addresses chronic stress, racial discrimination, and racism to reduce the fear of not being listened to must become a priority.

## Conclusion

This study highlights experiences of maternal traumatic experiences, health inequities, and structural racism. Including Black women in research, recruiting providers of color, and including nonhealth care providers who are advocates is critical to improving the health care experiences for Black women.^11,12^

## Significance

Persistent racial disparities indicate that clinical interventions alone cannot reduce racial disparities in infant and maternal mortality, because birth outcomes, like overall health, are the product of one's environment, opportunities, and experiences. In this article, we used content analysis to analyze the themes of the presentation made by five Black community members on a 2020 Juneteenth panel, a holiday celebrating the emancipation of those who had been enslaved in the United States. Participants unpacked the traumatic experiences of health inequities and structural racism on Black families and diverse caregivers. Four major themes emerged from the discussion: (1) the matrix of domination, (2) specific oppressions of the health care system, (3) empowerment reconceptualized, and (4) dimensions of racism.

## Abbreviations Used

BGLO: Black Greek Letter Organizations
CRT: critical race theory
KBEN: Kansas Birth Equity Network
SBHI: Sisters and Brothers for Healthy Infants
